# Influence of Amphibian Antimicrobial Peptides and Short Lipopeptides on Bacterial Biofilms Formed on Contact Lenses

**DOI:** 10.3390/ma9110873

**Published:** 2016-10-26

**Authors:** Magdalena Maciejewska, Marta Bauer, Damian Neubauer, Wojciech Kamysz, Malgorzata Dawgul

**Affiliations:** 1 Department of Inorganic Chemistry, Faculty of Pharmacy, Medical University of Gdansk, Gdansk 80-416, Poland; maciejewska.kj@gmail.com (M.M.); bauerm@gumed.edu.pl (M.B.); dneu@gumed.edu.pl (D.N.); kamysz@gumed.edu.pl (W.K.); 2 Pharmaceutical Laboratory, Avena SJ, Osielsko 86-031, Poland

**Keywords:** antimicrobial peptides, lipopeptides, biofilm, ocular infections, contact lenses

## Abstract

The widespread use of contact lenses is associated with several complications, including ocular biofilm-related infections. They are very difficult to manage with standard antimicrobial therapies, because bacterial growth in a biofilm is associated with an increased antibiotic resistance. The principal aim of this study was to evaluate the efficacy of antimicrobial peptides (AMPs) in eradication of bacterial biofilms formed on commercially available contact lenses. AMPs were synthesized according to Fmoc/tBu chemistry using the solid-phase method. Minimum inhibitory concentration (MIC) and minimum biofilm eradication concentration (MBEC) of the compounds were determined. Anti-biofilm activity of the antimicrobial peptides determined at different temperatures (25 °C and 37 °C) were compared with the effectiveness of commercially available contact lens solutions. All of the tested compounds exhibited stronger anti-biofilm properties as compared to those of the tested lens solutions. The strongest activity of AMPs was noticed against Gram-positive strains at a temperature of 25 °C. Conclusions: The results of our experiments encourage us toward further studies on AMPs and their potential application in the prophylaxis of contact lens-related eye infections.

## 1. Introduction

Contact lenses (CL) are one of the most popular biomaterials and represent a suitable surface for bacterial colonization in the eye, leading to ocular infection. Approximately 140 million people worldwide wear CL for vision correction [1]. The widespread use of CL is associated with a risk of complications, including CL-related bacterial infections [2]. The ability of different species of bacteria to adhere to the surface of biomaterials resulting in biofilm formation plays an important role in pathogenesis of CL-associated infections. Microbial cells inhabiting a three dimensional structure of biofilm have been reported to be up to 1000 times more resistant to antibiotics than their planktonic forms [3].

The surface of the eye is constantly exposed to numerous environmental factors, including a frequent adhesion of microorganisms. Majority of the environmentally-introduced microorganisms are constantly removed from the surface of the eye through the natural host defense mechanisms, such as tears, corneal nerves, epithelial cells, keratocytes, interferons, and innate immunity cells. The primary line of defense against ocular infection is provided by mechanical barriers, eyelids, and eyelashes. The blinking action of the eyelids removes most foreign particles and microorganisms from the eye and renews the tear film. Tears are one of the crucial elements of the eye’s defense system. They keep the eye lubricated and free from foreign bodies by flushing action. The tears’ film also provides an effective antimicrobial system capable of reducing the number of bacteria in vivo.

The tear fluid is composed of water, electrolytes, lipids, mucins and proteins (immunoglobulins, lysozyme, lactoferrin, lipocalin, and lacritin) [4,5]. A combination of lysozyme and lactoferrin has been found to be highly effective in eradicating staphylococci [6]. 

The most common ocular infections associated with CL are bacterial conjunctivitis, contact lens acute red eye (CLARE), contact lens peripheral ulcer (CLPU), infiltrative keratitis (IK), and microbial keratitis (MK). *Staphylococcus aureus*, *S. epidermidis*, *Streptococcus pneumonia*, *S. pyogenes*, *Haemophilus influenzae*, *Enterococcus* spp. *Moraxella* spp. *Escherichia coli*, *Serratia marcescens*, *Pseudomonas aeruginosa*, and *Proteus mirabilis* are the most common etiological factors associated with CL-related infections [1,7,8].

Handling CL, not complying with the hygienic procedures, replacement frequency and a poor quality of material, inadequate care solutions, and CL case compliance are major factors of CL contamination. There is also a strong correlation between CL extended wear and corneal infection and inflammation [9]. Extended wear of CL promotes enhanced adhesion of bacteria and consequently the development of corneal infection [10].

The current standard treatment of bacterial eye infections relies on conventional antibiotic therapy. However, the widespread use of broad-spectrum systemic antibiotics in ocular infection therapies resulted in an increase in resistance among the bacteria and induction of antibiotic-resistant strains. A significant number of *S. aureus* and coagulase-negative staphylococci, as well as *P. aeruginosa* associated with ocular infection, have been strongly antibiotic-resistant [11,12]. The standard therapy is of limited effectiveness in abatement of biofilm infection. 

Antimicrobial peptides (AMPs) have been found to exhibit activity against a wide range of bacteria responsible for ocular infections. These naturally occurring antimicrobial agents have been examined as therapeutic antibiotics. As a part of the innate immune system, AMPs can be applied to prevent the formation of biofilm as well as to eradicate mature structures. The mechanism of action of AMPs is based on the interaction with the cell membrane and carries a low risk of microbial resistance. AMP may be an alternative to conventional antibiotic treatment in ocular infection.

The most significant naturally occurring AMPs in the eye are human cathelicidin LL-37, as well as α- and β-defensins [4,5]. These compounds are secreted into tears by corneal and conjunctival epithelial cells and demonstrate a broad spectrum of antimicrobial activity. In response to inflammatory agents, neutrophils infiltrating the ocular surface supply tear fluid with additional LL-37 and α-defensins. *In vitro* studies confirmed antimicrobial activity of human β-defensins against methicillin-resistant *S. aureus* and *P. aeruginosa* in ocular infection [13]. The LL-37 is also produced by mast cells. The peptide significantly prevents from *S. epidermidis* adhesion and biofilm formation on the biomedical materials [5,13]. *In vitro* studies have shown that LL-37 is also effective in *P. aeruginosa* biofilm eradication [14]. Another AMP of tear fluid is psoriasin (S100A7). It is produced by cornea, conjunctiva, and lacrimal glands and has been demonstrated to be highly potent against *E. coli* [15].

Currently, several innovative strategies to optimize AMPs anti-biofilm activity are under evaluation with the aim to using them as a new class of antibiotics in the treatment of ocular infection. Direct topical AMPs application, production of AMP’s derivatives and peptidomimetics, construction of specifically-targeted AMPs (STAMP), or immobilization of AMPs directly onto the surface of biomaterials are some of the examples [16,17]. Several in vitro and in vivo studies have been performed to investigate a group of AMPs as potential anti-biofilm agents for ocular infection therapies [17,18,19,20,21].

The principal aim of this work was to evaluate the group of antimicrobial peptides with regard to their potential use for eradication of bacterial biofilms formed on CL.

## 2. Results

### 2.1. Minimum Inhibitory Concentration of the AMPs

The AMP*s* under study exhibited antimicrobial activity towards all of the tested bacteria, but their efficacy varied depending on the strain (Table 1). Pexiganan turned out to be the most effective peptide against planktonic bacteria. Bacterial growth was inhibited after exposure to pexiganan at concentrations of 1–8 µg/mL. Short synthetic lipopeptides also effectively inhibited the growth of all tested strains (4–32 µg/mL). The remaining peptides showed a comparable activity towards Gram-positive strains (8–32 µg/mL), while application of significantly higher concentrations of the compounds was required to inhibit the growth of Gram-negative strains (128–512 µg/mL). 

### 2.2. Anti-Biofilm Activity of Antimicrobial Peptides at Different Temperatures

The results of the tests with CL differed from those obtained for bacteria living in a planktonic form (Table 2 and Table 3). MBEC tests have shown that citropin 1.1, the weakest antimicrobial agent against planktonic cells, was the most potent anti-biofilm agent. It was the only compound that eliminated the living bacteria from the surface of CL at both temperature**s**. The MBEC values ranged from 16–64 µg/mL for Gram-positive strains. Biofilms formed by *E. coli* were eradicated after exposure to citropin 1.1 at concentration**s** of 128–256 µg/mL. *P. aeruginosa* was eliminated from the lens surface after application of citropin 1.1 at concentration of 512 µg/mL. Additionally, temporin A eliminated the bacteria at that concentration. The remaining peptides were inactive towards *P. aeruginosa* cultured on CL. Promising results were obtained with lipopeptide Pal-KK-NH_2_. The compound eradicated biofilms formed by Gram-positive bacteria at concentrations of 32–64 µg/mL below 25 °C and at 32–512 µg/mL at 37 °C. *E. coli* was eliminated by exposure to the compound at concentration of 256 µg/mL at both temperatures. The remaining compounds exhibited a moderate anti-biofilm activity. Surprisingly enough, pexiganan, the most potent antimicrobial agent in the MIC test, showed a rather feeble anti-biofilm activity. With certain strains and compounds the activity varied depending on the temperature. In many cases the MBEC values obtained for AMPs were 2–4 times lower at 25 °C than at 37 °C.

### 2.3. Efficacy of the Contact Lens Solutions against Biofilms

The commercial contact lens solutions were more effective after a single application at 37 °C (Table 4). This was observed for three out of four tested solutions against *S. pyogenes* and *E. coli*, and for one solution in case of *P. aeruginosa*. The CL solutions were active at both temperatures against biofilms formed on the lens surface by *S. aureus*, *S. epidermidis*, and *S. pneumoniae*. They were ineffective against *E. coli* and *S. pyogenes* cultured on lenses at 25 °C. The most difficult to eliminate from the surface of lenses was *P. aeruginosa*. It could not be eradicated after exposure to all solutions at 25 °C and to three out of four solutions at 37 °C.

## 3. Discussion

Biomaterials associated infections constitute a major barrier to the long-term use of medical devices and remain a serious therapeutic problem [17,22,23]. Bacterial growth in a form of biofilm is associated with an increased antibiotic resistance as compared to the growth under planktonic conditions [24]. 

CL represent an ideal surface for bacterial colonization in the eye. CL-related infections are relatively rare, but can pose severe vision-threatening complications. Standard treatment for most bacterial ocular infection is primarily empiric with broad-spectrum antibiotics. Unfortunately, the inadequate use of antibiotics leads to the development of resistant strains [25,26,27].

An alternative approach to the therapeutic problem of biofilm-associated infection would be the development of a new generation of AMP-based drugs [28,29]. In this study we confirmed antibacterial activity of the AMPs against common strains responsible for ocular infections. 

In the MIC test the most potent antimicrobial agent was pexiganan, an analogue of the naturally occurring magainin 2. Its strong antimicrobial activity has been reported by numerous studies [30,31,32]. The results clearly show the high effectiveness and broad-spectrum activity of pexiganan [33]. Potential therapeutic applications of the peptide and further development of its analogues are under way [34,35,36]. Although the compound has shown a broad spectrum of antimicrobial activity against planktonic cells of various bacterial strains, pexiganan turned out to be a weak anti-biofilm agent against structures formed on CL. This supports the finding that bacteria organized in a biofilm can exhibit a reduced susceptibility to AMPs. In our previous study reference bacterial strains susceptible to conventional antibiotics in their planktonic form were highly resistant once cultured as biofilms on polystyrene surfaces [32,37].

Both tested short lipopeptides also exhibited a high antimicrobial activity against all of the tested strains in the MIC test. Previous studies on Palm-KK-NH_2_ showed its strong activity against clinical strains of Gram-positive cocci including those resistant to antibiotics [38]. Pal-KK-NH_2_ demonstrated its ability to prevent a vascular graft biofilm formation in the rat model of staphylococcal infections. Moreover, efficiency of the lipopeptide was enhanced upon combination with standard drugs (e.g., vancomycin) [39]. Pal-KK-NH_2_ was also effective against a majority of strains in the biofilm form, while Pal-RR-NH_2_ showed a slightly lower activity. In our previous study the compounds were effective against biofilms formed by clinical isolates of *S. aureus* cultured on polystyrene [40]. 

Two amphibian peptides, citropin 1.1, naturally produced by *Litoria citropa* [41], and temporin A, primarily isolated from *Rana temporaria* [42], were highly effective against planktonic cultures of Gram-positive bacteria [43,44,45,46]. In the previous studies, temporin A also exhibited a strong activity against Gram-positive bacteria, including vancomycin-resistant strains [47]. Effectiveness of the compound was confirmed in animal models of infection caused by *S. epidermidis* [48] and *S. aureus* [49]. Research conducted by Simonetti et al. confirmed that temporin A was effective in MRSA-infected chronic wounds in mice [50]. Citropin 1.1 was also extensively studied. Most recently, it was found to be a potent anti-biofilm agent against *S. aureus* strains collected from patients with atopic dermatitis [51]. Moreover, the previous studies confirmed that the peptide demonstrates a synergy with rifampicin and minocycline against *S. aureus* biofilm [52]. In another study, citropin 1.1 showed a high efficiency in prevention of catheter-related infections caused by *S. aureus* [52]. Our results have shown that both compounds eliminated *P. aeruginosa* from CL, in contrary to the remaining peptides and applied CL solutions. Citropin 1.1 was the most effective compound in the study against biofilms formed by all of the tested strains.

The obtained results indicate that AMPs possess a potential for the development as a therapeutic and prophylactic agents against biofilm-related infections, especially those associated with Gram-positive pathogens.

In recent years a variety of peptides were investigated for their potential in the prevention of eye infections. For instance, Nos–Barbera et al. demonstrated positive effects of a cecropin-melittin hybrid peptide topically applied in a pseudomonas keratitis model in rabbits [18]. Willcox et al. developed antimicrobial cationic peptide coatings by covalent immobilization of melimine onto CL. Melimine is a hybrid of the active regions of two antimicrobial peptides: protamine (from salmon sperm) and melittin (from bee venom). Covalently-attached melimine significantly reduced clinical manifestations of CLARE in the *P. aeruginosa* guinea pig model [17]. *In vivo* melimine coatings demonstrated a significant reduction of CLPU incidence in the rabbit model [19]. The broad-spectrum antimicrobial activity of melimine was also confirmed in a human clinical trial, where melimine-coated lenses turned out to be safe for use during wear and retained a high antimicrobial activity after wear [20]. Other studies have shown that the melimine coating was heat-stable, non-toxic to mammalian cells in vitro, and did not alter the physical dimensions of CL [17,19,21].

In this study, we investigated peptides according to their potential application as CL solutions additives. All peptides eliminated Gram-positive bacteria from the surface of lenses, while only temporin A and citropin 1.1 showed some activity against Gram-negative bacteria. All of the tested CL solutions were ineffective against *S. pyogenes* and *P. aeruginosa* when cultured once at 25 °C. Three out of four applied solutions were also inactive towards *E. coli*. Similarly to previous studies, we have now confirmed that the efficacy of CL disinfecting solutions are dependent on the type of the bacterial strain [53,54,55]. The results of our study indicate that *P. aeruginosa* was the most resistant strain to the antimicrobial action of all of the tested fluids, a finding consistent with those of other studies [56,57]. 

Our research indicates that the susceptibility to antimicrobials of bacteria cultured on the lens surfaces is dependent on the temperature. For some strains and the tested AMPs, the anti-biofilm activity was stronger at 25 °C, while the tested lens solutions exhibited stronger activity at 37 °C. As room temperature is commonly recommended for the storage of CL, the use of commercial CL solutions appears to be rather inadequate for the prevention of ocular infections in CL users.

A harmony of proper hygiene and care habits of CL can effectively prevent contamination with pathogenic microorganisms [58]. Handling of CL, insufficient hand washing practice, and failure of some preservative systems are major sources of contamination implicated in the development of CL-associated infections [59,60,61]. According to the report of Turner et al., 44% of CL users do not wash their hands before handling, significantly increasing the risk of introducing pathogenic microorganisms into the eye [62]. The CL storage case and solutions may also be potential reservoirs for microorganisms responsible for ocular infections [5]. For example, studies exploring bacterial bioburden in a CL care system confirmed that storage case contamination is much more common than associated CL contamination [63,64,65]. The presence of pathogens in a storage case and in CL solutions significantly increase the risk of their transfer to the lens surface and, ultimately, onto the surface of the eye. 

Our data reveal that AMPs might be promising antibacterial additives to CL solutions. However there are certain limitations and issues which need to be considered. The long-time stability of compounds in a water solution needs to be examined, but first of all the evaluation of toxicity towards human eye cells needs to be carried out. In the previous study tested amphibian peptides turned out to be safe towards HaCaT cells at their microbiologically-active concentrations. In the same assay lipopeptide Pal-KK-NH_2_ exhibited high toxicity towards human keratinocytes [66]. Considering the potential toxicity and the results of the present study, the most interesting candidates for further examination are citropin 1.1 and temporin A. Encouraging results for other AMPs have been previously reported. Mannis et al. evaluated a synthetic cecropin analogue (Shiva-11) as an antibacterial agent in CL solutions. They demonstrated that it was effective against *P. aeruginosa*, *S. epidermidis* and *S. aureus* in a buffered saline containing a CL [67]. In an extensive study of the Shiva-11, Gunshefski et al. revealed that the peptide has a wide range of antimicrobial activity in vitro against human clinical ocular pathogens [68]. 

Sousa et al. examined the effectiveness of a cecropin analogue (D_5_C) against *P. aeruginosa* and compared it with the antimicrobial effect of commercial CL solutions. The investigators demonstrated that D_5_C substantially enhanced antimicrobial activity of the disinfecting solutions [69]. 

The results obtained in this study are consistent with those previously reported. AMPs show a broad-range antimicrobial activity and are potent anti-biofilm agents. Based on our results and the literature data, we came to the conclusion that AMPs show a considerable potential for the development of therapeutic and prophylactic antimicrobials against CL related infections. Further research is needed to establish the safety of ophthalmic application of the tested AMPs, as well as to optimize the composition of CL solutions, to ensure stability of the applied AMPs. Effective products, in combination with education on hygiene recommendations, will contribute to minimizing the risk of eye infections.

## 4. Materials and Methods 

### 4.1. Bacterial Strains and Culture Conditions

All selected strains were obtained from the Polish Collection of Microorganisms (Polish Academy of Science, Wroclaw, Poland). Four Gram-positive and three Gram-negative strains linked with CL-related infections lenses were tested (Table 5). The microorganisms were cultured in a Mueller Hinton Broth II (MHB, Biocorp, Warsaw, Poland) overnight, at 37 °C.

### 4.2. Antimicrobial Peptides

All of the peptides were synthesized in the Department of Inorganic Chemistry (Medical University of Gdansk, Gdansk, Poland). These were:

Pexiganan: GIGKFLKKAKKFGKAFVKILKK-NH_2_
Citropin 1.1: GLFDVIKKVASVIGGL-NH_2_
Temporin A: FLPLIGRVLSGIL-NH_2_
Lipopeptides: Palm-KK-NH_2_ and Palm-RR-NH_2_ (Palm–hexadecanoic acid residue)

### 4.3. Contact Lenses and Contact Lens Solutions

The CL and CL solutions used are commercially available. One-day CL (1-Day Acuvue Moist) containing Etafilcon A were obtained from Johnson and Johnson Vision Care (Jacksonsville, FL, USA). Four popular CL solutions were also tested (Table 6).

### 4.4. Peptide Synthesis

All of the peptides and lipopeptides were synthesized manually on polystyrene resin (Orpegen, Heidelberg, Germany) modified by a Rink Amide linker [70]. 9-Fluorenylmethoxycarbonyl (Fmoc) groups were used for protection of the α-amino groups of amino acids. Deprotection of the groups was carried out for 20 min using a 20% piperidine (Merck, Darmstadt, Germany) solution in *N*,*N*-dimethylformamide (DMF) (Honeywell, Seelze, Germany). Then the resin was washed in DMF and dichloromethane (DCM) (Chempur, Piekary Slaskie, Poland). To check the completion of the deprotection and acylation process, a chloranil test was performed. All amino acids (Orpegen, Heidelberg, Germany) were coupled using a DMF/DCM (1:1 *v/v*) mixture in the presence of coupling agents, 1-hydroxybenzotriazole (HOBt) (Orpegen, Heidelberg, Germany) and *N*,*N*-diisopropylocarbodiimide (DIC) (Merck, Darmstadt, Germany). The progress of each acylation was controlled by a chloranil test. The peptides were cleaved from the resin with a mixture of trifluoroacetic acid (TFA) (Merck, Darmstadt, Germany), water, triisopropylsilane (TIS) (Merck, Darmstadt, Germany), and phenol (Sigma Aldrich, Steinheim, Germany) as scavengers in the ratio 92.5:2.5:2.5:2.5 *v*/*v*. Once the sequences were obtained, the peptides were precipitated with cold diethyl ether (Chempur, Piekary Slaskie, Poland) and lyophilized. The crude products were purified and analyzed by reversed-phase high-performance liquid chromatography (RP-HPLC) in an acetonitrile-water (Sigma Aldrich, Steinheim, Germany) gradient containing 0.1% TFA. The identity of the peptides was confirmed by matrix-assisted laser desorption/ionization time of flight mass spectrometry (MALDI-TOF) and the counter-ion was determined by ion chromatography [71,72]. MS and HPLC analysis are included in Supplementary Materials.

### 4.5. Antimicrobial Activity Test Protocol

Peptide susceptibility tests on the reference bacterial strains were performed with the following procedures recommended by the Clinical and Laboratory Standard Institute (CLSI). The activity of five peptides was tested on the Mueller Hinton Broth II (Biocorp, Warsaw, Poland) using broth microdilution method on polystyrene 96-well plates (Kartell, Noviglio, Italy). Bacterial inoculum of ca. 5 × 10^5^ CFU/mL was added to each well and exposed to solutions of peptides at increasing concentrations (range 1–512 μg/mL). The plates were incubated overnight at 37 °C. MIC was assumed as the lowest concentration at which bacterial growth was no longer visible. All experiments were performed in triplicate.

### 4.6. Biofilm Assay

Biofilms of strains related with ocular infections were cultured on CL placed in polystyrene 24-well plates (Orange Scientific, Braine-l’Alleud, Belgium). Biomaterials were incubated at 37 °C in bacterial suspension in the Mueller Hinton Broth II (Biocorp, Warsaw, Poland) at initial inoculums of ca. 5 × 10^5^ CFU/mL. After 24 h of incubation all of the CL were rinsed three times with a sterile phosphate buffer (AppliChem, Darmstadt, Germany). The lenses were then transferred into new wells with fresh MHB II. One-day biofilms were exposed to a pre-determined range of peptides concentration. Plates with antimicrobial agents were incubated again for 24 h at 37 °C (optimal for bacterial growth) or 25 °C (room temperature-standard storage temperature for CL). Anti-biofilm activity of selected peptides was visualized using a cell-viability reagent, Resazurin (Sigma Aldrich, St. Louis, MO, USA). Upon contact with living cells the dye is metabolized by bacterial dehydrogenases resulting in the reduction of the blue resazurin to a pink resorufin. Positive controls contained CL immersed in bacterial suspensions without antimicrobials, while CL in a sterile culture medium served as negative controls. The minimum biofilm eradication concentration (MBEC) was read after a one-hour incubation with Resazurin (final concentration per sample = 0.005%) MBEC was determined as the lowest concentration at which the reduction of resazurin was lower or equal (10% ± 0.5%) as compared to positive (100%) and negative (0%) controls. All experiments were performed in triplicate.

### 4.7. Antimicrobial Activity of the Contact Lens Solutions

Effectiveness of the CL solutions against biofilms formed on CL was tested using the previously-described procedure, with the difference that in the place of peptides, different undiluted CL solutions were applied. Tested solutions were considered as active when they demonstrated the ability to reduce the number of living bacteria on the surface of CL by at least 90% (reduction of resazurin to lower or equal to (10% ± 0.5%) as compared to positive (100%) and negative (0%) controls). All experiments were performed in triplicate.

## Figures and Tables

**Table 1:** Minimum inhibitory concentration of the AMPs in μg/mL (μM).

Peptide	*S. aureus*	*S. epidermidis*	*S. pyogenes*	*S. pneumoniae*	*E. coli*	*P. aeruginosa*
Pexiganan	8	2	4	≤1	4	4
	(2.2)	(0.6)	(1.1)	(≤0.3)	(1.1)	(1.1)
Citropin 1.1	32	8	16	16	128	256
	(17.4)	(4.3)	(8.7)	(8.7)	(69.5)	(138.9)
Temporin A	8	4	4	8	256	512
	(4.9)	(2.5)	(2.5)	(4.9)	(157.5)	(315.1)
Palm-KK-NH_2_	8	4	16	8	8	16
	(10.8)	(5.4)	(21.7)	(10.8)	(10.8)	(21.7)
Palm-RR-NH_2_	8	4	16	4	16	32
	(10.1)	(5.0)	(20.1)	(5.0)	(20.1)	(40.3)

**Table 2:** Minimum biofilm eradication concentration (reduction of resazurin ≤10% ± 0.5%) at 37 °C in μg/mL (μM).

Peptide	*S. aureus*	*S. epidermidis*	*S. pyogenes*	*S. pneumoniae*	*E. coli*	*P. aeruginosa*
Pexiganan	>512	128	256	64	>512	>512
	(>141.5)	(35.4)	(70.8)	(17.7)	(>141.5)	(>141.5)
Citropin 1.1	64	16	32	32	256	512
	(34.7)	(8.7)	(17.4)	(17.4)	(138.9)	(277.8)
Temporin A	512	128	128	128	512	512
	(315.1)	(78.8)	(78.8)	(78.8)	(315.1)	(315.1)
Palm-KK-NH_2_	64	32	512	64	256	>512
	(86.6)	(43.3)	(693.0)	(86.6)	(346.5)	(>693.0)
Palm-RR-NH_2_	256	32	>512	32	512	>512
	(322.1)	(40.3)	(>644.1)	(40.3)	(644.1)	(>644.1)

**Table 3:** Minimum biofilm eradication concentration (reduction of resazurin ≤10% ± 0.5%) at 25 °C (μg/mL (μM)).

Peptide	*S. aureus*	*S. epidermidis*	*S. pyogenes*	*S. pneumoniae*	*E. coli*	*P. aeruginosa*
Pexiganan	256	16	128	64	512	>512
	(70.8)	(4.4)	(35.4)	(17.7)	(141.5)	(>141.5)
Citropin 1.1	32	16	32	64	128	512
	(17.4)	(8.7)	(17.4)	(34.7)	(69.5)	(277.8)
Temporin A	128	64	256	128	128	512
	(78.8)	(39.4)	(157.5)	(78.8)	(78.8)	(315.1)
Palm-KK-NH_2_	32	32	32	64	256	>512
	(43.3)	(43.3)	(43.3)	(86.6)	(346.5)	(>693.0)
Palm-RR-NH_2_	128	64	128	64	256	>512
	(161.0)	(80.5)	(161.0)	(80.5)	(322.1)	(>644.1)

**Table 4:** Efficacy of the contact lens solutions against biofilm at different temperatures (37 °C/ 25 °C).

*Lens Solution*	*S. aureus*	*S. epidermidis*	*S. pyogenes*	*S. pneumoniae*	*E. coli*	*P. aeruginosa*
A	+/+	+/+	−/−	+/+	+/−	−/−
B	+/+	+/+	+/−	+/+	+/+	+/−
C	+/+	+/+	+/−	+/+	+/−	−/−
D	+/+	+/+	+/−	+/+	+/−	−/−

“+” active; reduction of resazurin ≤10% ± 0.5%, “−“ inactive; reduction of resazurin ≥10% ± 0.5%.

**Table 5:** Reference bacterial strains.

Bacterial Group	Species	Number
Gram-positive	*Staphylococcus aureus*	ATCC 25923
	*Staphylococcus epidermidis*	ATCC 14990
	*Streptococcus pneumoniae*	ATCC 49619
	*Streptococcus pyogenes*	PCM 465
Gram-negative	*Escherichia coli*	ATCC 25922
	*Pseudomonas aeruginosa*	ATCC 9027

**Table 6:** Contact lens solutions and their contents.

Lens Solution	Components
A	Polyhexanide 0.0001%, Hydrolock (dexpanthenol and sorbitol), sodium phosphate, tromethamine, Poloxamer 407, disodium edetate.
B	Citrate, Tetronic 1304, aminomethylpropanol, sodium chloride, boric acid, sorbitol, disodium edetate, Polyquad (Polyquaternium) 0.001%, Aldox (myristamidopropyl dimethylamine) 0.0005%.
C	Hydroxyphosphate Alkyl, Boric Acid 0.03%, Disodium Edetate, Poloxamine 1%, Sodium Borate, Sodium Chloride, Polyaminopropyl Biguanide 0.0001%.
D	Boric Acid, disodium edetate, sodium borate, sodium chloride, DYMED (polyaminopropyl biguanide) 0.0001%, HYDRANATE (hydroxyalkylphosphonate) 0.03%, Poloxamine 1%.

## Supplementary Materials

MS and HPLC analysis are available online at www.mdpi.com/1996-1944/9/11/873/s1.
