# Supplementary material for: Influence of Amphibian Antimicrobial Peptides and Short Lipopeptides on Bacterial Biofilms Formed on Contact Lenses
Source: Materials (Basel). 2016 Oct 26;9(11):873. doi: 10.3390/ma9110873 (PMC5457276; doi:10.3390/ma9110873)

MS and HPLC analysis

Peptides:

1) Citropin 1.1
2) Pal-KK-NH_2
3) Pal-RR-NH_2
4) Pexiganan
5) Temporin A

Mass spectrometry

Equipment: Waters, ACQUITY QDa mass detector.
Range: 50-1250 m/z.
Positive scan, Cone voltage 15V, Sampling rate - 2 points/sec, capillary voltage 1.0 kV.

HPLC analysis

Equipment: Varian ProStar HPLC system,
Phenomenex, Luna® C18(2) column (100x3 mm, 5 µm, 100Å).
Mobile phase: Acetonitrile and water both containing 0.1 % of TFA (v/v).
Method: gradient of acetonitrile – 10-100% in 15 minutes. The data were collected for 16 min.
Detection: UV-Vis detector; absorbance at 214 nm.

Results

A\ MS analysis

A1: Citropin 1.1

Monoisotopic mass of the Citropin 1.1 is 1613.987 Da.

Table 1. Citropin 1.1.

z	Calculated m/z	Measured m/z
**1**	1614.99	-
**2**	808.00	808.72
**3**	539.00	539.60

A2: Pal-KK-NH_2

Monoisotopic mass of the Pal-KK-NH_2 is 511.44614 Da.

Table 2. Pal-KK-NH_2.

z	Calculated m/z	Measured m/z
**1**	512.45	512.63
**2**	256.73	-

Measured value of 1025.01 m/z refers to dimer – $[2M+2H]^{2+}$ (calculated value is 1024.91).

A3: Pal-RR-NH_2

Monoisotopic mass of the Pal-RR-NH_2 is 567.458 Da.

Table 3. Pal-RR-NH_2.

z	Calculated m/z	Measured m/z
**1**	568.47	568.63
**2**	284.74	285.02

A4: Pexiganan

Monoisotopic mass of the Pexiganan is 2475.630 Da.

Table 4. Pexiganan.

z	Calculated m/z	Measured m/z
**1**	2476,64	-
**2**	1238,82	1239.48
**3**	826,22	826.85
**4**	619,92	620.70
**5**	496,13	496.70
**6**	413,61	414.23
**7**	354,67	-
**8**	310,46	-
**9**	276,08	-
**10**	248,57	-

A5: Temporin A

Monoisotopic mass of the Temporin A is 1395.900 Da.

Table 5. Temporin A.

z	Calculated m/z	Measured m/z
**1**	1396,90	-
**2**	698,96	699.51

B\ HPLC analysis

B1: Citropin 1.1

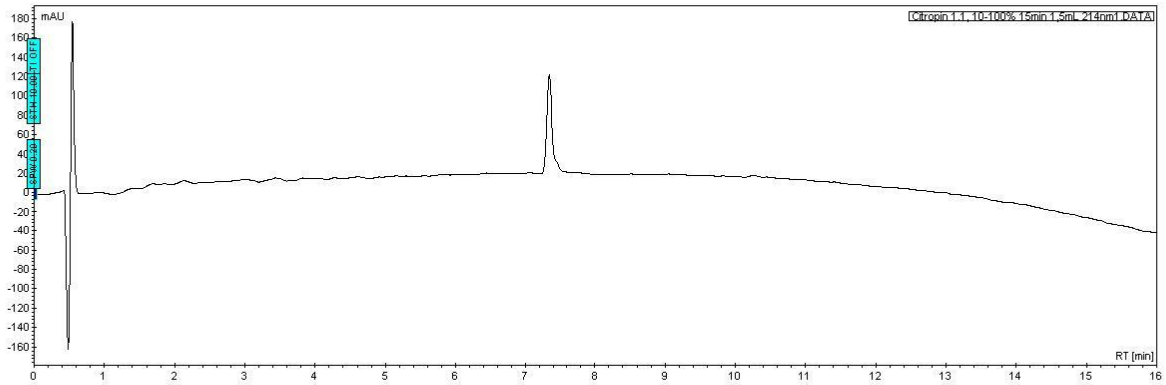

B2: Pal-KK-NH2

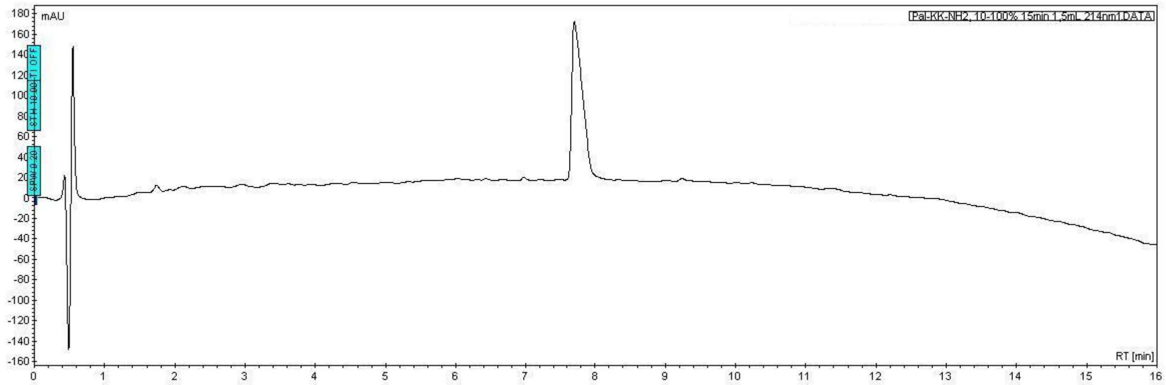

B3: Pal-RR-NH2

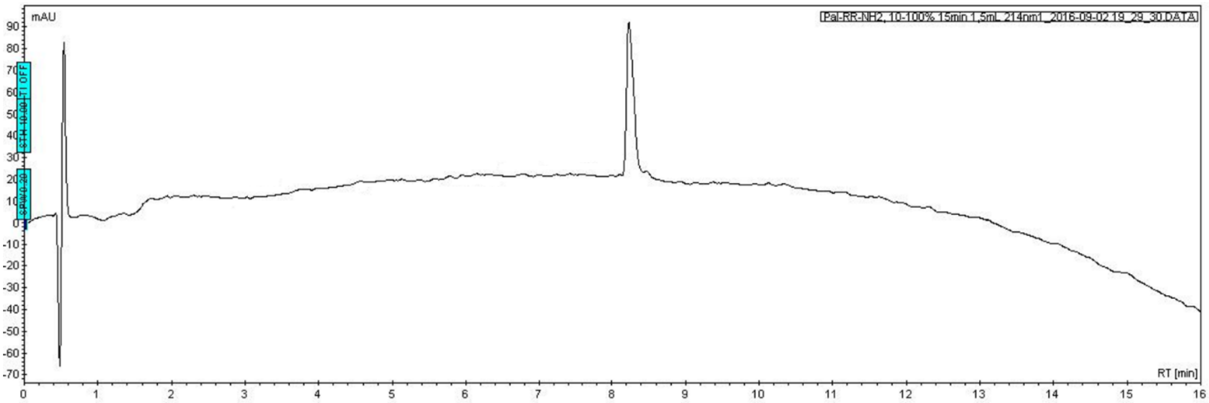

B4: Pexiganan

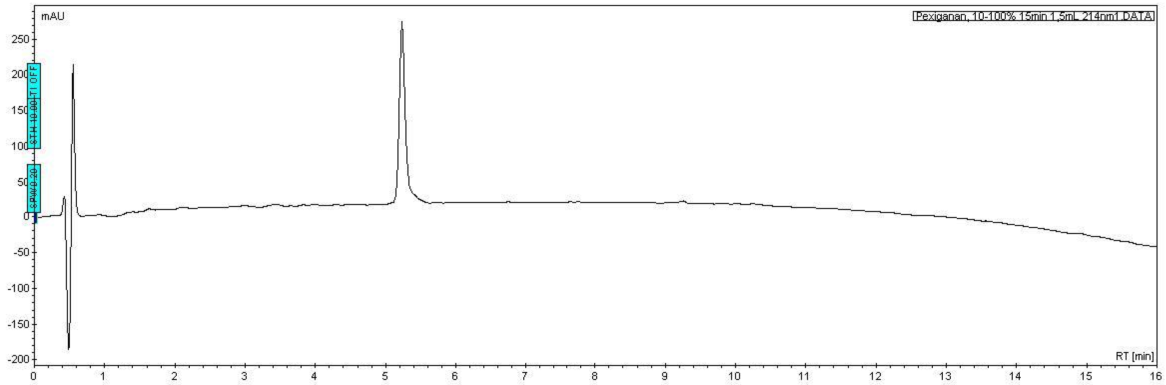

B5: Temporin A

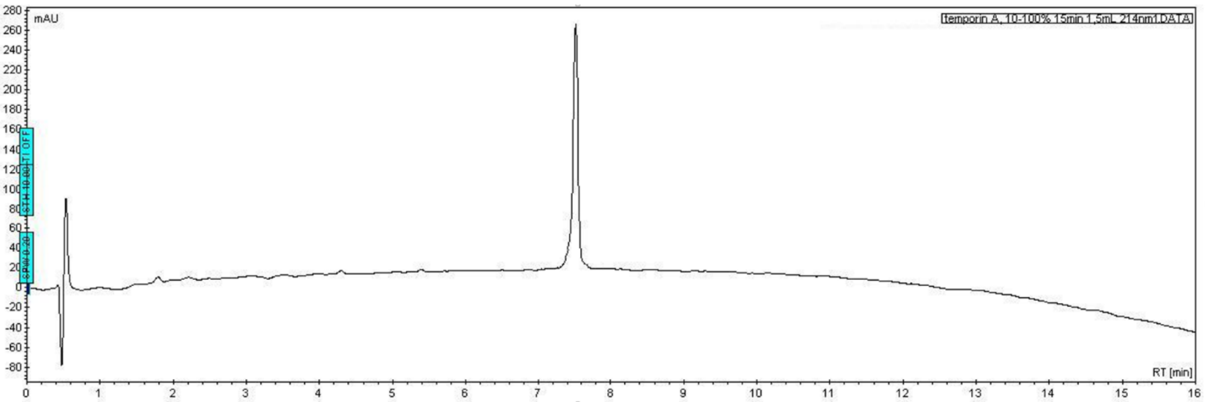

Supplement: Supplementary file 1 [file materials-09-00873-s001.pdf]
